# Novel Roles for Urokinase- and Tissue-Type Plasminogen Activators in the Pathogenesis of Mood Disorders

**DOI:** 10.3390/ijms26146899

**Published:** 2025-07-18

**Authors:** Amine Bahi, Sinclair Steele

**Affiliations:** 1 Basic Medical Sciences Department, College of Medicine, Ajman University, Ajman P.O. Box 346, United Arab Emirates; 2 Center of Medical & Bio-Allied Health Sciences Research, Ajman University, Ajman P.O. Box 346, United Arab Emirates; 3 Pathological Sciences Department, College of Medicine, Ajman University, Ajman P.O. Box 346, United Arab Emirates

**Keywords:** plasminogen activator system, neuronal plasticity, urokinase-type plasminogen activator (uPA), tissue-type plasminogen activator (tPA), therapeutic strategies, mood disorders, depression

## Abstract

This narrative review explores the intricate relationship between the plasminogen activator system (PAS), comprising urokinase-type plasminogen activator (uPA) and tissue-type plasminogen activator (tPA), and a range of neuropsychiatric disorders, including depression and anxiety. By synthesizing existing preclinical and clinical evidence, we clarify the roles of uPA and tPA in the pathogenesis and potential treatments of these conditions. This narrative review emphasizes their involvement in modulating neuronal plasticity, synaptic remodeling, and neurotransmitter systems, which are pivotal in maintaining brain function and behavior. Additionally, this review highlights key mechanisms by which these activators influence the neurobiological processes underlying mood and cognitive dysfunction. Critical analysis identifies areas of consensus, such as the role of plasminogen activators in neuroinflammation and stress responses, while also addressing gaps and controversies in the literature. The findings underscore the therapeutic potential of targeting the uPA/tPA system for innovative interventions. By offering a nuanced understanding of their contributions to mood disorders, this review aims to inspire future research toward developing novel, mechanism-based treatment strategies that harness the PAS’ capacity to restore neural homeostasis and improve patient outcomes.

## 1. The Plasminogen Activator System (PAS): A Brief Overview

uPA and tPA, while primarily recognized for their roles in fibrinolysis, exert a significant influence on neuronal plasticity and neurotransmission. This influence contributes to the pathogenesis of several mood disorders through diverse mechanisms, including modulation of brain-derived neurotrophic factor (BDNF) and other intricate signaling pathways. The involvement of uPA and tPA varies across different disorders, underscoring the complexity of their actions and the need for further research to clarify their precise roles in each specific condition. This section provides a foundational overview of the uPA and tPA system, encompassing their enzymatic functions, mechanisms of action, and primary physiological roles. This establishes a framework for understanding their involvement in neurological processes relevant to mood disorders.

### 1.1. Enzymatic Function and Mechanisms

uPA and tPA are serine proteases [1,2,3]. Their primary function is the conversion of plasminogen, an inactive zymogen, into plasmin, a highly active serine protease responsible for fibrinolysis [3,4]. This conversion is an important step in the breakdown of fibrin clots, essential for maintaining vascular patency, hemostasis, and tissue repair [3]. The activity of both uPA and tPA is tightly regulated, primarily through interactions with specific inhibitors, the most prominent being plasminogen activator inhibitor-1 (PAI-1) [3,5]. PAI-1 binds to uPA and tPA, forming inactive complexes that prevent plasmin generation [3]. The balance between PA activity and PAI-1 levels is important in maintaining hemostasis and preventing excessive fibrinolysis [6]. Further regulation occurs through receptor binding and other intricate mechanisms [4,5,6]. The specific interactions and regulatory mechanisms differ for uPA and tPA, leading to their distinct functional roles in various physiological processes. For example, uPA is often associated with cell surface-associated proteolysis [3], while tPA plays a more prominent role in the liquid phase [4].

### 1.2. Physiological Roles

**Figure 1:**
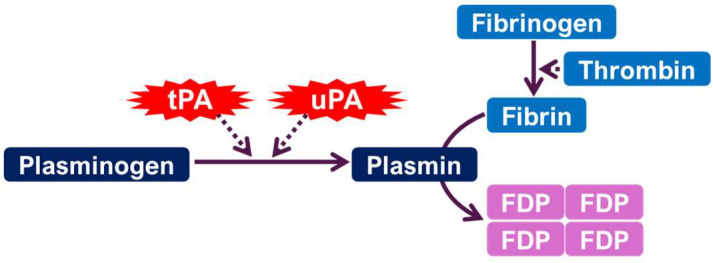
The plasminogen activating system. tPA: tissue-type plasminogen activator. uPA: urokinase-type plasminogen activator. FDP: fibrin degradation products.

Beyond their well-established roles in fibrinolysis, uPA and tPA are involved in a wide range of physiological processes. They are key components in both physiological and pathological tissue remodeling, including wound healing and tumor angiogenesis. These enzymes facilitate the breakdown of extracellular matrix (ECM) components, activate matrix metalloproteinases, and release proangiogenic molecules such as VEGF-A, thereby supporting tissue regeneration and vascular remodeling [3,7,8]. Their functions extend to processes such as ovulation and trophoblast invasion during pregnancy [3,9], demonstrating their involvement in regulated tissue breakdown and cellular migration (Figure 1). In the nervous system, uPA and tPA play an important role in synaptic plasticity [1,10], influencing the structural and functional remodeling of synapses that underly learning and memory [11]. The precise mechanisms by which uPA and tPA contribute to synaptic plasticity are being actively investigated; however, evidence suggests their involvement in processes such as long-term potentiation and long-term depression [1,10]. The dynamic regulation of uPA and tPA activity is essential for proper brain function, and dysregulation may contribute to neurological and psychiatric disorders.

## 2. Preclinical Studies: Animal Models of Depression and the uPA/tPA System

**Figure 2:**
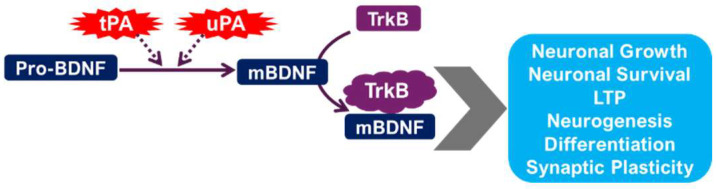
The role of the plasminogen activating system and BDNF maturation in neuronal survival, neuroplasticity, and synaptogenesis.

In this section, we review the intricate roles of uPA and tPA in the pathogenesis of depression. This review draws upon both preclinical and clinical studies to synthesize a comprehensive understanding of this complex relationship. The PAS, encompassing uPA, tPA, their inhibitors (PAI-1), and plasmin, is important in a multitude of physiological processes within the central nervous system (CNS) [12,13]. These processes include, but are not limited to, ECM remodeling, synaptic plasticity, and neuronal survival. Emerging evidence strongly suggests that dysregulation within the PAS, specifically involving imbalances in uPA and tPA activity and PAI-1 levels, contributes significantly to the development, progression, and treatment response in depressive disorders. We analyze the existing literature to build a detailed and nuanced understanding of this intricate interplay. This review delves into preclinical findings from animal models of depression, examining the effects of chronic stress, genetic manipulations, and the tPA/BDNF pathway. Subsequently, we analyze clinical studies investigating serum levels of uPA, tPA, and PAI-1 in depressed patients; explore the tPA/BDNF pathway in humans; and discuss the implications for treatment response (Figure 2). Finally, this review addresses the role of inflammation in this context and outlines therapeutic implications and future research directions.

### 2.1. Chronic Stress Models and the Plasminogen System

Preclinical research extensively employs chronic stress paradigms to induce depression-like behaviors in rodents, providing valuable insights into the underlying molecular mechanisms [14,15]. These models mimic aspects of human depression, allowing for researchers to investigate the effects of chronic stress on the brain and behavior. Commonly used chronic stress models include chronic unpredictable mild stress (CUMS), chronic restraint stress (CRS), and social defeat stress (SDS) [16,17,18,19,20]. These models induce a range of behavioral changes, such as anhedonia, reduced motivation, and increased anxiety, which resemble core symptoms of depression in humans. Importantly, studies using these models have revealed consistent alterations in the expression and activity of components of the PAS within brain regions critically implicated in the pathogenesis of depression, including the hippocampus and amygdala [21,22,23,24,25]. The hippocampus, a brain structure necessary for learning and memory, exhibits significant structural and functional changes in depression, and the amygdala, involved in processing emotions, is implicated in anxiety and fear responses.

Studies have demonstrated that chronic stress exposure leads to alterations in the balance of uPA and tPA activity, often manifested as an increase in PAI-1 levels [11,21]. This imbalance can disrupt normal ECM remodeling and synaptic plasticity within the hippocampus and amygdala. The increased PAI-1 inhibits the activity of both uPA and tPA, leading to reduced plasmin generation. Plasmin is a key protease involved in ECM degradation and remodeling, and its reduced activity can impair synaptic plasticity and neuronal survival. Furthermore, the specific changes observed in uPA and tPA expression and their functional consequences vary depending on the specific chronic stress model employed and the brain region examined. For instance, CUMS might induce different alterations in PAS components compared to CRS, and these alterations may be more pronounced in the hippocampus than in the amygdala [21]. The complexity of these interactions is being unraveled by the active and ongoing research into the precise mechanisms involved.

### 2.2. Genetic Manipulations of uPA and tPA

Further insights into the roles of uPA and tPA in depression-related behaviors have been gained through studies employing genetic manipulations in mice [21]. These studies involve creating transgenic mice with altered uPA or tPA expression levels. By modifying the genetic makeup of these animals, researchers can directly investigate the causal role of these proteins in depression-related phenotypes. Mice with either increased or decreased expression of uPA or tPA often exhibit alterations in their behavioral responses to stress, including changes in anxiety-like behaviors and depressive-like symptoms [21]. For example, mice with reduced uPA expression might show a blunted stress response, while mice with overexpressed tPA might exhibit increased anxiety-like behaviors. The observed behavioral changes highlight the importance of maintaining a balanced plasminogen system for normal emotional regulation and stress coping mechanisms. However, the precise mechanisms by which uPA and tPA dysregulation lead to these behavioral changes remain to be fully elucidated. These studies often focus on specific pathways and signaling cascades downstream of uPA and tPA activation, which impair ECM remodeling, synaptic plasticity, neuronal survival, and neurogenesis. Systemic inflammation, exemplified by gut dysbiosis, abdominal fat tissue, silicone breast implants, chronic sinusitis, and psychological stress, is associated with elevated levels of interleukin-6 (IL-6), tumor necrosis factor-alpha (TNF-α), and transforming growth factor-beta (TGF-β) [26,27,28,29,30,31]. These cytokines can cross the blood–brain barrier and promote endothelial dysfunction and neurovascular inflammation. IL-6, in particular, can induce PAI-1 expression via trans-signaling pathways in endothelial cells, thereby reducing the fibrinolytic activity of uPA and tPA [32,33]. Subsequent research will benefit from investigating potential interactions between uPA and tPA signaling pathways and their effects on neurotransmitter systems implicated in depression.

### 2.3. The tPA/BDNF Pathway in Depression

#### 2.3.1. Neurotrophic Factors and Synaptic Plasticity

**Figure 3:**
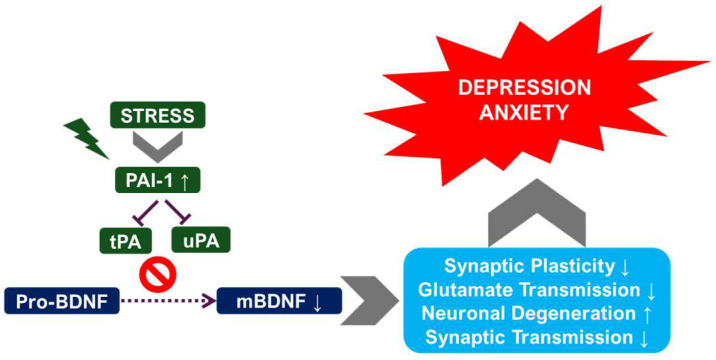
Alteration of functional BDNF and depression. ↑ increase; ↓ decrease.

Depression is associated with reduced levels of BDNF in several brain regions, including the hippocampus [34,35,36]. BDNF plays a pivotal role in neuronal survival, growth, and synaptic plasticity [37,38,39]. Reduced BDNF levels contribute to the neuronal atrophy and impaired synaptic plasticity observed in depression. The PAS may interact with BDNF signaling pathways, potentially influencing BDNF expression and activity [10,40]. uPA and tPA may modulate BDNF levels either directly or indirectly through their effects on synaptic plasticity [41]. For instance, alterations in tPA activity could affect the processing of proBDNF to mature BDNF [41], impacting BDNF’s neurotrophic effects on hippocampal neurons [42]. The disruption of this intricate balance could contribute to the neurobiological changes underlying depression. The observed alterations in dendritic spine density and synaptic function in depression may also be influenced by uPA and tPA activity [42,43], further highlighting the complexity of their roles (Figure 3).

The interplay between tPA and BDNF represents a significant area of investigation in depression research [12,44]. BDNF, a key neurotrophin, plays an important role in neuronal survival, synaptic plasticity, and neurogenesis. It exists in two main forms, both of which are active: proBDNF (precursor) and BDNF. tPA plays an important role in the conversion of proBDNF to BDNF [12,22,44]. Preclinical studies have consistently demonstrated that alterations in the tPA/BDNF pathway are implicated in the pathogenesis of depression [44,45]. Specifically, reduced serum levels of tPA, impairing the conversion of proBDNF to BDNF, have been observed in depressed animals [44]. This imbalance can lead to impaired synaptic plasticity, reduced neurogenesis, and increased neuronal apoptosis, which are neuropathological features of depression. Further research is underway to fully understand the complex interplay between tPA, BDNF, and proBDNF in different brain regions and their contributions to the development and maintenance of depressive symptoms.

#### 2.3.2. Clinical Studies: Investigating uPA and tPA in Human Depression

A number of clinical studies have investigated the relationship between serum levels and functional activity of PAS components and their association with major depressive disorder (MDD) in human populations [12,44]. These studies aim to evaluate whether alterations in these proteins could serve as reliable biomarkers or provide mechanistic insights into the pathogenesis of depression. While findings across studies vary, a recurring observation is the interplay between decreased protein levels and decreased enzymatic activity, which together may contribute to functional impairment of the tPA/BDNF axis. For instance, lower serum levels of tPA have been reported in depressed individuals [44], which may reflect a reduction in the enzymatic conversion of proBDNF to mature BDNF (BDNF), an essential step for supporting synaptic plasticity, neurogenesis, and neuronal survival.

Further supporting this pathway, several studies have shown a decreased BDNF/proBDNF ratio in patients with depression, suggesting impaired neurotrophin maturation and reduced neurotrophic signaling [12,44]. This biochemical imbalance mirrors preclinical findings and is increasingly recognized as a molecular hallmark of depressive pathology. In parallel, elevated levels of PAI-1, an endogenous inhibitor of both uPA and tPA, have been observed in patients with MDD, as shown in a recent systematic review and meta-analysis [46]. By binding to and inhibiting uPA and tPA, increased PAI-1 levels effectively suppress their catalytic activity, thereby disrupting downstream processes such as ECM remodeling, synaptic reorganization, and BDNF activation.

These overlapping mechanisms suggest that decreased PAS protein levels and decreased enzymatic activity represent two sides of the same coin, collectively leading to a dysfunctional neuroplasticity response in depression. However, despite these promising associations, the clinical literature remains inconclusive. Discrepancies among studies may arise from variability in sample size, patient heterogeneity (e.g., age, sex, medication status, comorbidities), depression subtypes (e.g., melancholic vs. atypical), and laboratory methods used to quantify protein levels and activity. Moreover, many of the available data are cross-sectional in nature, limiting the ability to infer causality or temporal dynamics [47,48,49].

To advance the field, future research should prioritize well-powered longitudinal studies that follow patients across different stages of illness and treatment [50,51,52]. Such designs will be critical to determine whether PAS component levels can predict depression onset, monitor disease progression, or serve as early indicators of treatment response. Additionally, stratifying patients by depression subtype or inflammatory profile may help clarify the role of the tPA/BDNF pathway in specific subpopulations. Ultimately, a deeper understanding of how PAS dysregulation affects neurobiological resilience may inform novel therapeutic strategies targeting the enzymatic machinery involved in neurotrophin activation.

#### 2.3.3. Treatment Response and the Plasminogen System

The potential link between the PAS and antidepressant treatment response has also been a focus of clinical research [44,45]. Some studies suggest that changes in PAS component levels may be associated with treatment outcome, indicating a potential role for the PAS in mediating the therapeutic effects of antidepressants. For example, a study investigating the response to paroxetine treatment in Alzheimer’s disease patients with depressive symptoms found an association between a specific SERPINE1 polymorphism (rs1799889, which affects PAI-1 expression) and treatment response [45]. This suggests that genetic variations influencing PAI-1 levels may influence the effectiveness of antidepressant treatment. Studies examining the changes in PAS component levels before, during, and after antidepressant treatment may shed light on the temporal dynamics of these changes and their relationship to treatment efficacy. Furthermore, investigating whether specific antidepressants differentially affect the PAS could provide important insights into the mechanisms of action of the different classes of antidepressants.

### 2.4. The Role of Inflammation and the uPA/tPA System in Depression

#### 2.4.1. Neuroinflammation and the Plasminogen System

The involvement of neuroinflammation in the pathogenesis of depression has gained considerable traction in recent years [12,53]. Neuroinflammation refers to the activation of immune cells within the CNS, leading to the release of inflammatory cytokines and other mediators that can damage neurons and disrupt brain function. The PAS is intricately linked to inflammatory processes, with uPA playing a significant role in mediating the migration and activation of immune cells [21,54]. In the context of depression, chronic low-grade inflammation within the brain may affect the expression and activity of uPA and tPA, contributing to the development and maintenance of depressive symptoms. For example, elevated levels of inflammatory cytokines can influence the expression of PAI-1, leading to an imbalance in the PAS and impaired ECM remodeling. This imbalance can disrupt synaptic plasticity and neuronal survival, contributing to the neuropathological changes observed in depression.

Moreover, systemic inflammation has been shown to disrupt the integrity of the blood–brain barrier (BBB), allowing for peripheral cytokines and immune mediators to enter the CNS and exacerbate neuroinflammatory responses. Disruption of the BBB can occur via multiple mechanisms, including tight junction breakdown, endothelial dysfunction, and cytokine-mediated permeability changes [55,56]. Inflammatory insults, such as LPS, TNF-α, and IL-6 can trigger these changes, heightening the sensitivity of the CNS to peripheral immune signals and amplifying PAS dysregulation [57,58]. This compromised barrier function enables greater trafficking of inflammatory cytokines, immune cells, and neurotoxic molecules, which may further suppress tPA and uPA activity via PAI-1 upregulation, impair BDNF maturation, and disrupt synaptic function. Ultimately, these changes contribute to a feed-forward loop of neuroinflammation, synaptic loss, and depressive pathology.

Further research is needed to elucidate the complex interplay between neuroinflammation, the PAS, and the development of depressive disorders. Understanding the precise molecular mechanisms involved may well lead to the development of novel anti-inflammatory therapies for depression.

#### 2.4.2. Inflammatory Markers and Treatment Response

Clinical research is increasingly exploring the relationship between inflammatory markers and the response to antidepressant treatment [59]. Some studies suggest that individuals with elevated levels of inflammatory markers may exhibit a poorer response to antidepressant medication [59]. This indicates that inflammation may influence the effectiveness of antidepressant treatment, possibly by interfering with the mechanisms through which antidepressants exert their therapeutic effects. This suggests that targeting inflammation, potentially through indirect modulation of the PAS, could be a promising therapeutic strategy for improving treatment outcomes in depression. Further studies will explore potential strategies for combining anti-inflammatory therapies with antidepressants to enhance treatment efficacy. It is important to investigate whether specific inflammatory markers are more strongly associated with treatment response than others and to explore the potential interaction effects between inflammatory markers and genetic factors.

### 2.5. Therapeutic Implications and Future Directions

#### 2.5.1. Targeting the Plasminogen System for Depression Treatment

The accumulating evidence from both preclinical and clinical studies suggests that manipulating the uPA and tPA system may offer therapeutic potential for depression [11]. However, significant challenges remain in translating these findings into effective clinical treatments. These would include identifying specific therapeutic targets within the PAS (e.g., uPA, tPA, PAI-1) and developing safe and effective interventions for depression that can selectively modulate their activity. This would involve developing drugs that specifically inhibit or enhance the activity of uPA, tPA, or PAI-1 or targeting the upstream signaling pathways that regulate their expression. Preclinical studies would test the efficacy and safety of these potential interventions in animal models of depression, followed by well-designed clinical trials to evaluate their effectiveness in human populations.

#### 2.5.2. Biomarker Discovery and Diagnostic Applications

The discovery of reliable biomarkers for depression is a major factor in improving diagnosis, predicting treatment response, and identifying individuals at high risk of developing depression [12,44]. Studies examining serum levels of uPA, tPA, PAI-1, and BDNF have shown them to be promising potential biomarkers. Validating these biomarkers and determining their clinical utility are goals that are being enthusiastically pursued. This includes large-scale studies comparing biomarker levels in depressed individuals and healthy controls as well as longitudinal studies to assess the stability and predictive value of these biomarkers over time. The development of accurate and reliable diagnostic tools based on these biomarkers would significantly improve the early detection and management of depression. Naturally, this would lead to earlier intervention and improved treatment outcomes.

#### 2.5.3. Limitations and Future Research Directions

The current literature on the role of uPA and tPA in depression presents several methodological challenges that require addressing in subsequent studies. These include inconsistencies in study designs (e.g., cross-sectional vs. longitudinal), variations in sample characteristics (age, sex, severity of depression, comorbid conditions), and differences in the methods used to measure uPA, tPA, PAI-1, and BDNF. Future studies should aim to standardize methods and use larger, more homogenous samples to improve the reliability and generalizability of the findings. This includes using well-validated assays for measuring PAS components and controlling for potential confounding factors, such as age, sex, and medication use. Careful consideration of pre-analytical variables, such as blood collection and processing techniques, is important in minimizing artifacts and ensuring accurate measurements.

While the evidence suggests a link between uPA/tPA dysregulation and depression, the precise mechanisms underlying this relationship are still being elucidated. Accordingly, this requires investigating the interactions between PAS, neuroinflammation, synaptic plasticity, and neurotransmitter systems in depression. Studies using advanced techniques, such as in vivo imaging and genetic manipulation, can help unravel the complex interplay between these factors. Investigating the role of specific signaling pathways downstream of uPA and tPA activation is also key in the understanding of their contribution to depression-related neuropathology.

Bridging the gap between preclinical and clinical findings is essential for translating research into effective clinical treatments for depression. Clearly, validating preclinical findings in human populations and investigating the clinical utility of modulating the plasminogen system for depression treatment are extremely important. This includes conducting well-designed clinical trials to test the efficacy and safety of potential interventions targeting the PAS. It is also important to investigate whether different clinical subtypes of depression respond differently to interventions targeting the PAS. Accordingly, this might involve stratifying patients based on clinical characteristics or biomarkers to identify subgroups that could benefit most from such treatments.

#### 2.5.4. Conclusion: The Plasminogen System—A Promising Avenue for Depression Research

This comprehensive literature review has highlighted the accumulating evidence implicating the uPA and tPA system in the pathogenesis of depression. Preclinical studies using animal models have provided valuable insights into the alterations in the expression and activity of these proteins in response to stress and in association with depression-like behaviors. Clinical studies have offered some support for these findings but highlight the need for more research. Methodological limitations, including variations in study designs and measurement techniques, have contributed to the lack of definitive conclusions. Future research will benefit from standardizing methods, using larger sample sizes, and elucidating the precise mechanisms underlying the relationship between the PAS and depression. Targeting the plasminogen system through biomarker discovery and the development of novel therapeutic interventions represents a significant and promising avenue for future research in depression. The potential to improve diagnosis, predict treatment response, and develop novel therapies makes this area of research highly significant for improving the lives of individuals affected by depression.

## 3. Roles of uPA and tPA in Anxiety Disorders and PTSD

In this section, we examine the roles of uPA and tPA in anxiety disorders, including generalized anxiety disorder (GAD) and post-traumatic stress disorder (PTSD), based on both preclinical and clinical studies. Emerging evidence suggests the involvement of the PAS in a wider range of physiological and pathophysiological processes, including modulation of mood and cognitive functions [60]. We analyze the existing literature, comparing and contrasting findings, highlighting areas of agreement and disagreement, and identifying research gaps.

### 3.1. uPA’s Role in Anxiety and PTSD

#### 3.1.1. Preclinical Studies

Preclinical research using animal models has yielded significant insights into uPA’s potential role in modulating anxiety and stress-related behaviors. We demonstrated that hippocampal uPA overexpression in rats mitigated stress-induced anxiety- and depression-like behaviors [60]. This effect was correlated with increased levels of BDNF in the hippocampus, suggesting a potential mechanism through which uPA exerts its anxiolytic effects. The study employed several behavioral tests, including the marble burying test, open field test, elevated plus maze test, sucrose splash test, tail suspension test, and forced swim test [60], providing a comprehensive assessment of anxiety- and depression-related behaviors. The strongly positive correlation between hippocampal uPA levels and BDNF, together with the negative correlation between uPA mRNA and anxiety/depression parameters, further supports uPA’s involvement in mood regulation [60]. However, the precise mechanisms underlying uPA’s action in the hippocampus and its interaction with BDNF continue to be elicited.

Other preclinical studies have investigated uPA’s role in different contexts. For instance, research on uPA in the context of intracerebral hemorrhage (ICH) [61] has shown that uPA can ameliorate oedema and improve outcomes in rat models of ICH [61]. While not directly addressing anxiety or PTSD, these findings highlight uPA’s broader impact on brain function and the potential for its therapeutic manipulation in various neurological conditions. The study [61] also noted the upregulation of BBB protein expression following uPA treatment, suggesting potential benefits in conditions where BBB integrity is compromised, a factor that could indirectly influence anxiety and stress responses. Further research is needed to explore the potential interplay between uPA’s effects on the BBB and its impact on anxiety- and stress-related behaviors.

#### 3.1.2. Clinical Studies

Relatively few clinical studies have directly investigated uPA’s role in anxiety and PTSD. The paucity of clinical data in this area represents a significant research gap. The preclinical findings regarding uPA’s anxiolytic and antidepressant effects [60] warrant further investigations with human subjects. Clinical trials will assess the efficacy and safety of uPA-based therapies for anxiety and PTSD. Such trials should consider various factors, including dosage, administration route, and potential side effects, as well as patient characteristics like age, gender, and severity of the disorder. The correlation observed in animal models between uPA and BDNF [60] suggests a potential biomarker for treatment response in humans.

### 3.2. tPA’s Role in Anxiety and PTSD

#### 3.2.1. Preclinical Studies

Preclinical research on tPA’s role in anxiety and PTSD is limited compared to the research on uPA. While tPA is primarily known for its role in fibrinolysis and its use in stroke treatment [62,63,64,65,66], some studies suggest it may have neuroprotective effects [67]. Yepes proposed that tPA, in addition to its proteolytic function, may interact with N-methyl-D-aspartate receptors (NMDARs) and activate the mammalian target of the rapamycin (mTOR) pathway, leading to increased glucose uptake in neurons and adaptation to metabolic stress [67]. This non-proteolytic function of tPA could have implications for neuronal survival and function in conditions involving stress and anxiety. However, the link between this neuroprotective mechanism and anxiety/PTSD is still being investigated.

Moreover, tPA’s role in the context of stroke, while not directly related to anxiety or PTSD, could indirectly influence these conditions. Studies showing that thrombus permeability influences the efficacy of tPA treatment in ischemic stroke [66] highlight the complexity of tPA’s effects on the brain. Indirectly, the impact of stroke on mood and cognitive function could contribute to anxiety and PTSD development, and this warrants further investigation. The use of tPA in stroke treatment also presents potential side effects, including hemorrhagic transformation [68], which could further complicate the assessment of tPA’s role in anxiety and stress-related conditions.

#### 3.2.2. Clinical Studies

There are few clinical studies examining tPA’s direct role in anxiety and PTSD. The majority of clinical research on tPA focuses on its use as a thrombolytic agent in acute ischemic stroke [62,63,64,65,66,69,70,71,72,73]. Although some studies suggest sex-specific differences in tPA’s effectiveness in stroke [69,71], these findings are not directly applicable to anxiety or PTSD. The potential neuroprotective effects of tPA [67] in the context of stress and anxiety require further investigation through well-designed clinical trials.

### 3.3. Plasminogen Activator Inhibitor-1 (PAI-1) and Anxiety Disorders

PAI-1 is the primary physiological inhibitor of both tPA and uPA [74]. Although there is limited direct evidence linking PAI-1 to anxiety and PTSD, its involvement in inflammatory processes [74,75,76] and its influence on cellular behavior suggest a potential indirect role. While PAI-1 itself is not a proinflammatory molecule, inflammatory states are associated with elevated PAI-1 levels due to cytokine-mediated transcriptional activation. Specifically, the PAI-1 promoter is responsive to IL-6, TNF-α, and TGF-β, all of which are elevated in chronic inflammation [77,78]. Chronic inflammation is associated with various mental health disorders [74], and this cytokine-driven upregulation of PAI-1 may indirectly contribute to the development or worsening of anxiety and PTSD. Further research is needed to explore this potential link, including studies investigating PAI-1 levels in patients with anxiety disorders and PTSD. The complexity of PAI-1’s actions, including its interaction with vitronectin and the urokinase receptor (uPAR) [74], further complicates the understanding of its potential role in anxiety and PTSD.

### 3.4. Comparative Analysis of uPA and tPA

While both uPA and tPA belong to the PAS and are involved in fibrinolysis, their roles in anxiety and PTSD appear to differ. Preclinical studies have provided more evidence suggesting an anxiolytic role for uPA, particularly in the hippocampus [60], whereas the evidence for tPA’s direct involvement in anxiety and PTSD is less robust. tPA’s primarily known for its role in fibrinolysis and its clinical use in stroke treatment [62,63,64,65,66,69,70,71,72,73], while its potential neuroprotective effects [67] could indirectly impact anxiety and PTSD. The limited clinical data for both uPA and tPA in anxiety and PTSD highlight a significant research gap. Future studies could compare the effects of uPA and tPA on anxiety- and stress-related behaviors, both preclinically and clinically, to better understand their individual contributions and potential synergistic effects.

### 3.5. Limitations and Futur Directions

This section highlights significant gaps in our understanding of uPA’s and tPA’s roles in anxiety and PTSD. The majority of existing research focuses on preclinical studies using animal models, which may not fully translate to human conditions. Clinical studies are limited, particularly regarding uPA’s role. Furthermore, the complex interplay between the PAS, inflammation, and the neurobiological mechanisms underlying anxiety and PTSD is at an early investigative stage. Ideally, future research should prioritize the following:

- Conducting well-designed clinical trials to investigate the efficacy and safety of uPA-based therapies for anxiety and PTSD.
- Exploring the potential neuroprotective effects of tPA in the context of stress and anxiety, considering both its proteolytic and non-proteolytic functions.
- Investigating the role of PAI-1 in anxiety and PTSD, focusing on its potential contribution to inflammation and its interaction with uPA and tPA.
- Developing more sophisticated animal models that better reflect the complexity of human anxiety disorders and PTSD.
- Employing multi-modal approaches that integrate behavioral, neuroimaging, and molecular data to understand the mechanisms underlying the effects of uPA and tPA on anxiety and stress-related behaviors.

### 3.6. Conclusion

While the existing literature suggests a potential role for uPA and tPA in anxiety and PTSD, primarily based on preclinical findings, significant research gaps remain. Further investigation is crucial to translate the promising preclinical data into effective clinical therapies. A comprehensive understanding of the complex interactions within the PAS and its influence on the neurobiological underpinnings of anxiety and PTSD is essential for the development of novel and targeted treatments for these debilitating disorders. Future research should prioritize clinical trials and multi-modal approaches to address these research gaps.

## 4. Summary of Key Findings

This review demonstrates the significant involvement of uPA and tPA in the pathogenesis of several mood disorders, including anxiety and depression (Table 1). While the precise mechanisms vary across disorders, consistent themes emerge, including alterations in BDNF levels, impaired synaptic plasticity, and contributions to neuroinflammation. The evidence suggests that uPA and tPA are not merely bystanders in these conditions but rather active participants, modulating key neurobiological processes. The findings highlight the complexity of these systems and the interconnectedness of various biological pathways in these conditions. However, it is important to note that the promising research in this area that aims to fully elucidate the precise roles of uPA and tPA in each disorder is still evolving.

## 5. General Conclusion

The findings discussed in this review have significant therapeutic implications. Modulation of uPA and tPA activity or expression could represent a novel therapeutic target for mood disorders. The development of such therapeutic strategies presents several challenges. The complexity of the uPA/tPA system, its involvement in multiple physiological processes, and its varied roles across different disorders necessitate a cautious and nuanced approach. Further research is crucial to delineate the precise mechanisms by which uPA and tPA contribute to each disorder, identify specific biomarkers of dysregulation, and develop targeted therapeutic interventions with minimal side effects. Future studies should focus on understanding the interactions between uPA/tPA and other signaling pathways, such as BDNF and inflammatory cascades, to develop more effective treatments for mood disorders. The development of specific inhibitors or activators of uPA and tPA, tailored to specific disorders and pathways, is a promising avenue for future research.

## Figures and Tables

**Table 1:** Summary of preclinical and clinical studies linking tPA, uPA, and PAI-1 to mood disorders, with associated brain regions, effects, and behavioral or clinical outcomes. ↑ increase; ↓ decrease.

Component	Study Type	Species/Subject	Mood Disorder Type	Effect Direction	Brain Region (If Known)	Behavioral/Clinical Marker	Ref
tPA	Preclinical	Mouse	Depression	↓ tPA linked to ↓ BDNF, impaired synaptic plasticity	Hippocampus	BDNF levels, behavior tests	[44]
tPA	Preclinical	Rat	Depression	Impaired proBDNF to BDNF conversion	Hippocampus	BDNF processing	[41]
tPA	Clinical	Human	Depression	↓ tPA levels in MDD patients	Serum	ELISA, clinical scales	[12]
tPA	Clinical	Human	Depression	↓ BDNF/proBDNF ratio correlates with ↓ tPA	Serum	BDNF ratio, clinical diagnosis	[44]
tPA	Preclinical	Rat	Stroke-linked anxiety	Possible neuroprotection via mTOR and NMDARs	Global brain	Glucose uptake, neuroprotection	[67]
uPA	Preclinical	Mouse	Depression	Reduced uPA & blunted stress response	Amygdala, hippocampus	Behavioral tests	[21]
uPA	Preclinical	Rat	Anxiety/Depression	uPA overexpression with ↓ anxiety and ↑ BDNF	Hippocampus	BDNF levels, behavioral tests	[60]
uPA	Clinical	Human	Depression	Not directly studied but inferred role	Serum	NA	[12]
PAI-1	Preclinical	Mouse	Depression	↑ PAI-1 with ↓ tPA/uPA activity	Hippocampus	Plasmin activity, behavioral outcomes	[21]
PAI-1	Clinical	Human	Depression	↑ PAI-1 in MDD patients	Serum	Meta-analysis data	[46]
PAI-1	Clinical	Human	Depression (AD + depression)	SERPINE1 polymorphism affects response to SSRIs	Genetic/Serum	Genotyping, clinical outcomes	[45]
PAI-1	Clinical	Human	Anxiety/PTSD	↑ PAI-1 via cytokines IL-6, TNF-α, TGF-β	Endothelium/CNS	Cytokine assays, inferred role	[78]

## Abbreviations

The following abbreviations are used in this manuscript:

ALI	Acute Lung Injury
BBB	Blood–Brain Barrier
BDNF	Brain-Derived Neurotrophic Factor
CKD	Chronic Kidney Disease
CNS	Central Nervous System
CRS	Chronic Restraint Stress
CUMS	Chronic Unpredictable Mild Stress
ECM	Extracellular Matrix
FDP	Fibrin Degradation Products
GAD	Generalized Anxiety Disorder
ICH	Intracerebral Hemorrhage
LTD	Long-Term Depression
LTP	Long-Term Potentiation
mBDNF	Mature Brain-Derived Neurotrophic Factor
MDD	Major Depressive Disorder
mTOR	Mammalian Target of Rapamycin
NMDAR	N-Methyl-D-Aspartate Receptor
PAI-1	Plasminogen Activator Inhibitor-1
PAS	Plasminogen Activator System
proBDNF	Precursor Brain-Derived Neurotrophic Factor
PTSD	Post-Traumatic Stress Disorder
SDS	Social Defeat Stress
SERPINE1	Serpin Family E Member 1 (gene encoding PAI-1)
TBI	Traumatic Brain Injury
tPA	Tissue-Type Plasminogen Activator
uPA	Urokinase-Type Plasminogen Activator
